# Structured reporting has the potential to reduce reporting times of dual-energy x-ray absorptiometry exams

**DOI:** 10.1186/s12891-020-03200-w

**Published:** 2020-04-16

**Authors:** Su Hwan Kim, Lara M. Sobez, Judith E. Spiro, Adrian Curta, Felix Ceelen, Eric Kampmann, Martin Goepfert, Raphael Bodensohn, Felix G. Meinel, Wieland H. Sommer, Nora N. Sommer, Franziska Galiè

**Affiliations:** 1 Department of Radiology, University Hospital, LMU Munich, Munich, Germany; 2 Munich Transplant Center, University Hospital, LMU Munich, Munich, Germany; 3 Department of Internal Medicine III, University Hospital, LMU Munich, Munich, Germany; 4 Department of Radiation Oncology, University Hospital, LMU Munich, Munich, Germany; 5 Department of Diagnostic and Interventional Radiology, Paediatric Radiology and Neuroradiology, University Medical Center Rostock, Rostock, Germany

**Keywords:** X-rays, Clinical decision-making, Decision trees, Quality improvement, Bone density

## Abstract

**Background:**

In recent years, structured reporting has been shown to be beneficial with regard to report completeness and clinical decision-making as compared to free-text reports (FTR). However, the impact of structured reporting on reporting efficiency has not been thoroughly evaluted yet. The aim of this study was to compare reporting times and report quality of structured reports (SR) to conventional free-text reports of dual-energy x-ray absorptiometry exams (DXA).

**Methods:**

FTRs and SRs of DXA were retrospectively generated by 2 radiology residents and 2 final-year medical students. Time was measured from the first view of the exam until the report was saved. A random sample of DXA reports was selected and sent to 2 referring physicians for further evaluation of report quality.

**Results:**

A total of 104 DXA reports (both FTRs and SRs) were generated and 48 randomly selected reports were evaluated by referring physicians. Reporting times were shorter for SRs in both radiology residents and medical students with median reporting times of 2.7 min (residents: 2.7, medical students: 2.7) for SRs and 6.1 min (residents: 5.0, medical students: 7.5) for FTRs. Information extraction was perceived to be significantly easier from SRs vs FTRs (*P* <  0.001). SRs were rated to answer the clinical question significantly better than FTRs (*P* <  0.007). Overall report quality was rated significantly higher for SRs compared to FTRs (*P* <  0.001) with 96% of SRs vs 79% of FTRs receiving high or very high-quality ratings. All readers except for one resident preferred structured reporting over free-text reporting and both referring clinicians preferred SRs over FTRs for DXA.

**Conclusions:**

Template-based structured reporting of DXA might lead to shorter reporting times and increased report quality.

## Introduction

In previous years, there have been efforts by several international radiological societies to improve the quality of radiological reports through structured reporting [1–5]. Several studies have shown that structured reports (SR) tend to be more complete and may contribute to better clinical decision-making compared to conventional free-text reports (FTR) [6–13]. Structured reporting may be especially useful in highly standardized exams, e.g. cranial MRI scans in multiple sclerosis patients [14] or videofluoroscopic exams [15]. Structured reporting has also been shown to be beneficial in examinations with complex criteria which need to be provided for referring physicians (e.g. in oncologic imaging for staging of rectal cancer [2], hepatocellular carcinoma [13], pancreatic carcinoma [3] or diffuse large B-cell lymphoma [16]). However, there is also an ongoing debate on potential disadvantages of structured reporting, such as the risk of oversimplification [17], distraction by additional software [18], and the challenge of integrating structured reporting tools into the clinical workflow [19].

Another aspect of the debate is the matter of time with suspected prolonged reporting times for SRs, especially in the transition phase from current free-text reporting practices to structured reporting [1, 20]. One concern is that productivity might be reduced because of more time spent looking at the template rather than the study [21]. To date, there are only a few studies that evaluated the impact of structured reporting on reporting times, one for leg-length discrepancy measurements [22], one in the area of emergency radiology [23] and another one on mammography and ultrasound in breast cancer patients [24] – all of them showing the potential of improved reporting times when using structured reporting. Nevertheless, as a change in workflow from the current practice of creating FTRs using speech recognition systems to creating SRs is likely to take some time, a good study design is necessary to evaluate the potentially time-saving effect of structured reporting. It may therefore be advisable to focus on a very standardized exam with limited complexity such as the dual x-ray absorptiometry exam (DXA). Although there is a continuous discussion on whether alternative types of bone densitometry measurements, such as quantitative computer tomography (QCT), might be superior to DXA [25, 26], DXA is still viewed as the international gold standard for bone mineral density measurements by many authors [27–30].

The International Society for Clinical Densitometry (ISCD) has published a best practice guideline for DXA reporting [31] and provided an adult DXA sample report that follows these official positions. A recent study revealed that major errors in DXA reporting are very common and their occurrence can be reduced drastically when a reporting template in accordance with these ISCD suggestions is implemented [32]. In addition, the latest 2019 ISCD Official Positions specify detailed requirements of baseline and follow-up DXA reports each [33].

However, it has yet to be shown if structured reporting of DXA exams does not only improve report accuracy but is also time-saving.

Therefore, we aimed at comparing reporting times of SRs compared to FTRs for DXA while at the same time evaluating completeness of information, facilitation of information extraction and overall quality.

## Materials and methods

### Patient selection and study design

Retrospectively, 26 DXA scans with acquisition dates between April 1st and June 30th, 2016 were randomly selected from the 100 most recent scans in our institutional radiology information system. Images of the femur and lumbar spine were acquired during the same session, using Lunar Prodigy (GE Healthcare, Chicago, USA).

For most patients, the referring physicians requested the DXA for clinical surveillance of osteoporosis and in some cases to confirm osteoporosis. Exams were included in the study if both the femur and the lumbar vertebral column had been measured on the same day and at least one previous scan acquired at our hospital was available for comparison. Our patient sample consisted of 3 men (mean age: 76 years, min. 67, max. 90) and 23 women (mean age: 70 years, min. 35, max. 86). The study was approved by the institutional review board. Informed consent was waived by the review board as data were analyzed anonymously as part of our department`s internal quality management program.

### Sample size calculations

Sample size calculations were performed based on expected differences of reporting times for DXA exams of conventional FTRs compared to SRs. Since there have been no prior studies on this issue, mean reporting times of FTRs for DXA by the residents that had been generated during the training phase were applied as a rough estimate. Based on the average reporting time of 318 s ± 75 SD, a reduction of 20% in reporting time for a SR was considered as a relevant effect size. Assuming those differences between both reporting types, a sample size of *N* = 44 (22 per group) would be required to achieve a power of 80% at a level of significance α = 0.05. To account for the possibility of overestimating the reduction in reporting time for SRs, an extra of 8 reports were added, leading to a final sample size of *N* = 52 (26 per report type). This sample size was used for both residents and medical students, adding up to 104 reports in total.

### Template for structured reporting

**Fig. 1:**
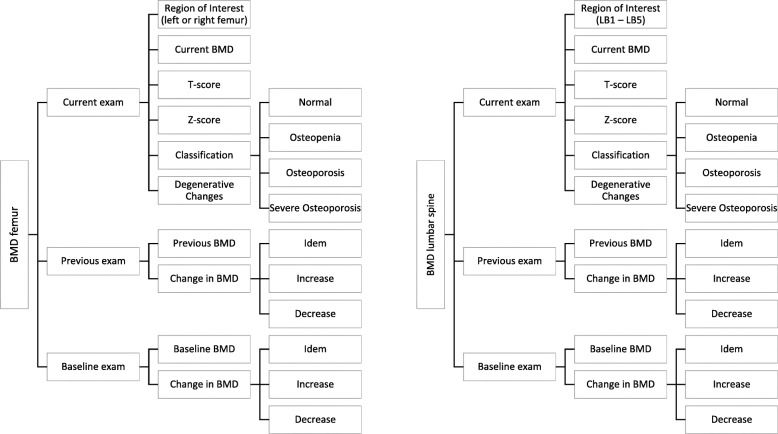
Structure of DXA reporting template: The template was created based on international guidelines by the International Society for Clinical Densitometry and the National Osteoporosis Foundation, including all standard elements of a densitometry report such as the t-score, z-score and World Health Organization criteria. BMD: bone mineral density

**Fig. 2:**
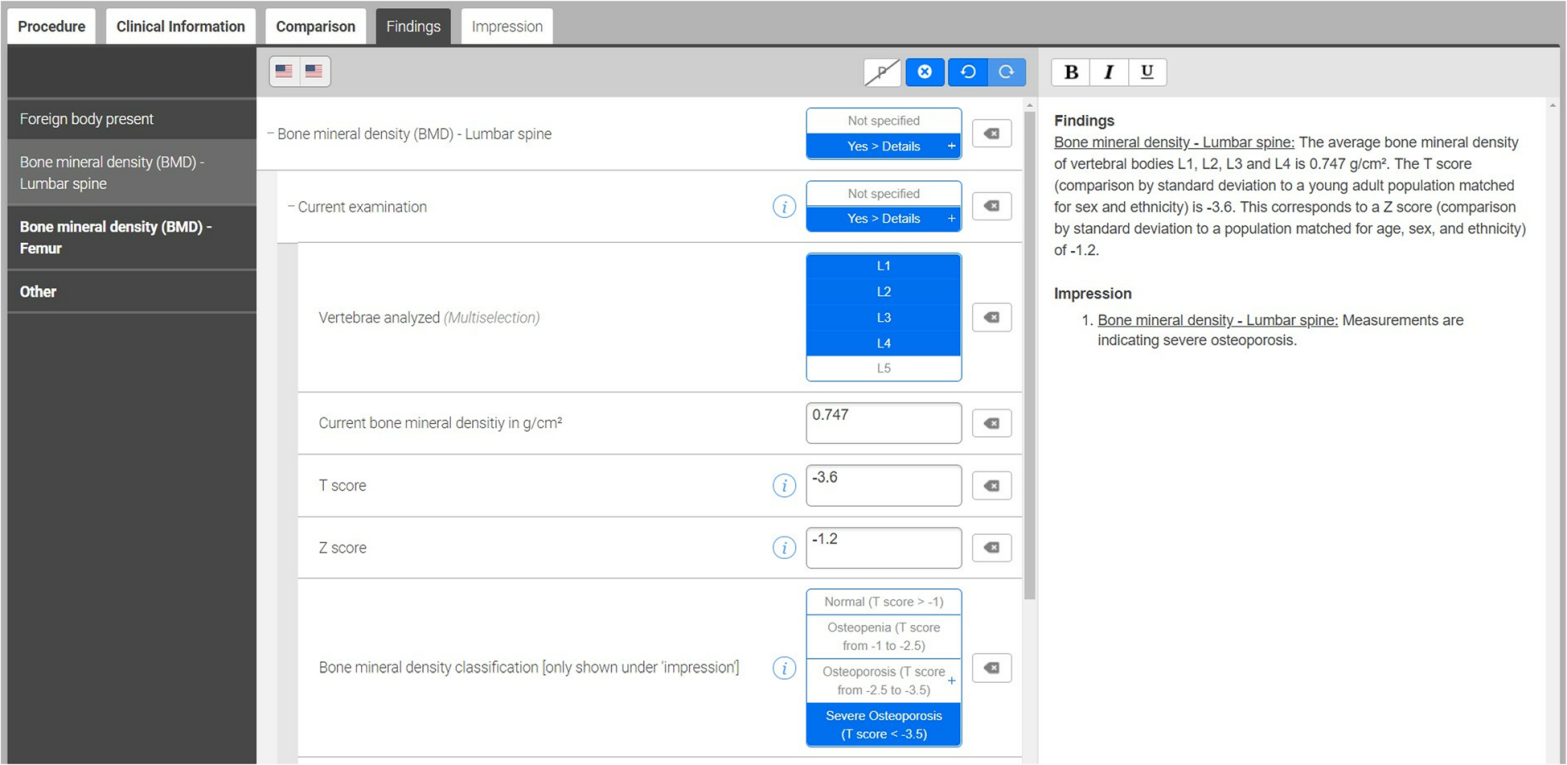
Screenshot of DXA reporting template: When pre-defined options are selected or values are entered into text fields, the final report text is generated automatically [retrieved from Smart Radiology software, “X-ray bone density” template, on 18 Aug 2018]

The reporting template we implemented was designed using software from Smart Reporting GmbH (Munich, Germany). This online software allows users to design templates for creating SRs. It consists of point-and-click menus representing a pre-defined decision tree. The template was created based on international guidelines by the ISCD and the National Osteoporosis Foundation (NOF), including all standard elements of a densitometry report such as the t-score, z-score and WHO criteria [31]. From these user entries, the template generates complete sentences automatically. The report can then be easily exported by using a one-click copy-and-paste button (see Figs. 1 and 2).

### Creation of radiologic reports

The FTRs and SRs were created by two radiology residents and two final-year medical students from a single academic medical center in Munich, Germany. They were randomly assigned to two cohorts with each cohort consisting of one resident and one medical student. The residents had 21 and 40 months of experience in radiology each and had previously dictated 25–50 and less than 25 DXA reports, respectively. The medical students had 2 and 4 months of dedicated experience in radiology and had no prior experience in creating DXA reports. Since our dictation software is personalized and only available for physicians, the medical students had to type their FTRs, whereas the residents dictated theirs.

**Fig. 3:**
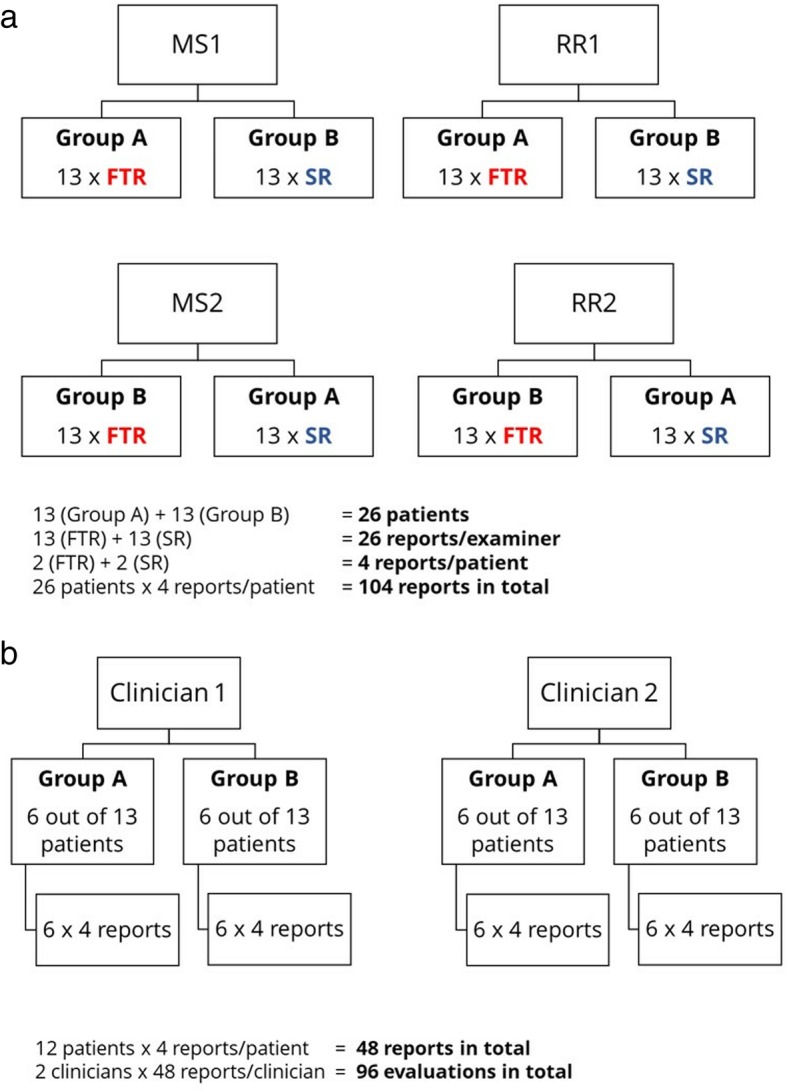
Study design: A DXA scans of 26 patients were randomized and examined by two medical students and two radiology residents; each reader created either a SR or FTR for each scan B 48 out of 104 reports were evaluated by two clinicians specializing in internal medicine; MS: medical student, RR: radiology resident, FTR: free text report, SR: structured report

In an initial training-phase the principle of DXA scans was reviewed and a demonstration of how the SRs can be created using the pre-defined online template was given. Each reader created only one type of report (SR or FTR) for each of the 26 patients. We used a cross-over design so that no reader would create more than one report per patient and recall-bias could be avoided. The patients were randomized into two groups of 13 (see Fig. 3a). Then both cohorts began creating reports for the same group of patients. After completing 13 reports, the readers switched to creating the other report type for the remaining 13 patients. They documented the time it took from the moment a DXA scan was opened in the imaging software program until the report was saved.

### Evaluation of the reports

To ensure that potentially reduced reporting times did not come at the cost of lower report quality, we asked two clinicians specializing in internal medicine to rate the reports (2 and 8 years of experience with patients who require DXA exams).

**Fig. 4:**
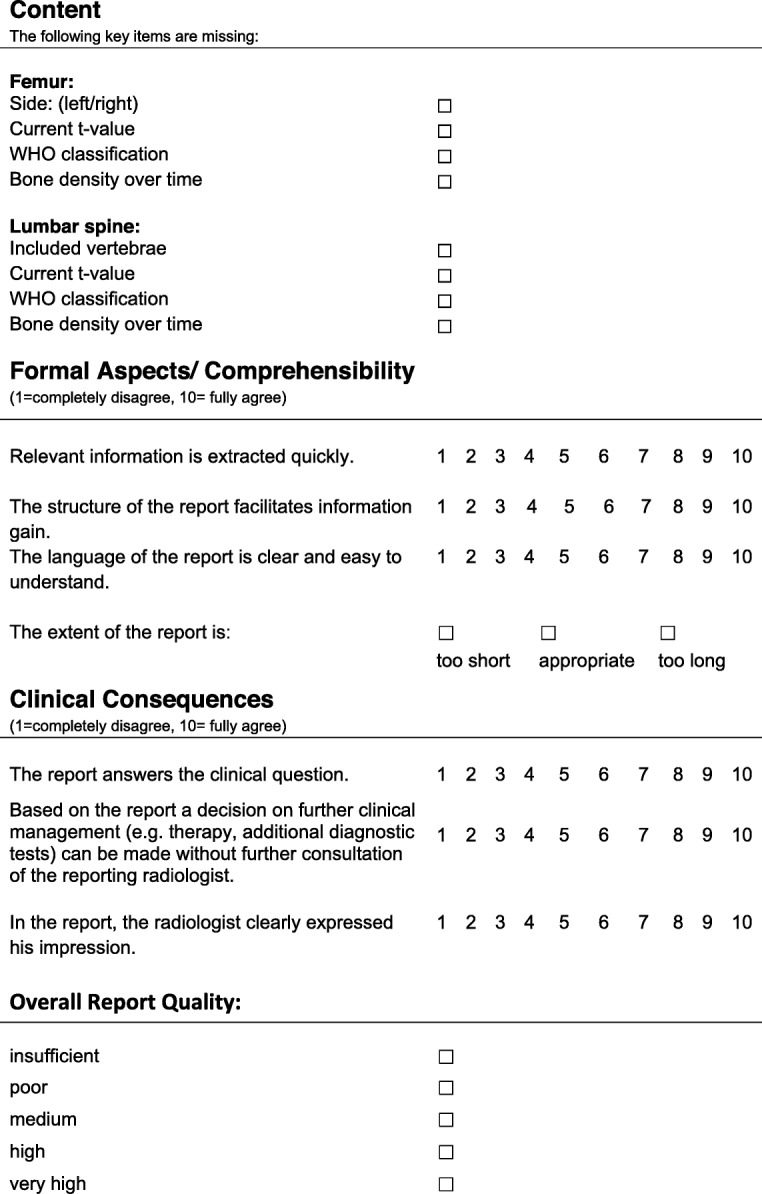
Report evaluation questionnaire: This figure shows the report evaluation questionnaire using checkboxes and 10-point Likert scale questions for the three main categories (content, clarity and clinical consequences)

From each of the two patient groups, six patients were randomly selected, resulting in a total of 12 patients, whose reports were evaluated by the clinicians. There were four reports for each of the 12 patients, adding up to 48 reports in total (see Fig. 3b). These anonymized reports contained the clinical question, a unique report ID, age and gender of the patient. SRs and FTRs were uniformly formatted. The evaluating physicians were blinded to report type and to the level of the reader’s experience who had created the report. A standardized questionnaire for DXA report evaluation was provided based on an extensive literature review [7, 10, 17, 24, 34–37] to ascertain the most important features of a good radiology report. Among others, content, comprehensibility and clinical relevance were rated on a 10-point Likert scale (see Fig. 4).

### Reader’s survey

After completing the reports, the four readers answered an anonymous online survey containing Likert scale and open-ended questions regarding the template and their opinions on structured reporting vs. free-text reporting (see Table in Additional file 3).

### Statistical analysis

Data are reported as medians with interquartile range (IQR) and minimum and maximum for ratings on a 10-point Likert scale and as frequencies and percentages for categorical items. The reporting times for each report type were compared separately for residents and medical students (Mann-Whitney-U-test) as voice-recognition software was unavailable to the medical students. To compare reporting times between residents and medical students for the template-based SRs the Wilcoxon signed rank test for paired data was used since one resident and one medical student always created a SR for the same patient. The report ratings by referring physicians were compared using the Wilcoxon signed rank test (Likert scale items and overall rating). The level of significance was defined at α <  0.05. SPSS Version 20 was used for all statistical calculations.

## Results

### Reporting times

Both the residents and the medical students required less time for SRs than for FTRs (see Table 1 and Table in Additional file 1). The median reporting time of residents amounted to 4.96 min for FTRs, compared to 2.71 min for SRs, which corresponds to a reduction of 45.4% (*P* <  0.001). The effect was even more distinct for the medical students who required 64.4% less time for SRs than for FTRs (median for FTRs: 7.53 min; median for SRs: 2.68 min; *P* <  0.001).

**Table 1:** Reporting times in minutes of radiology residents and final-year medical students for DXA exams

	Median	IQR	Min.	Max.	P
**OVERALL AVERAGE** (*n* = 104)					
Residents (*n* = 52)					
FTR	4.96	3.98–6.15	2.77	7.45	
SR	2.71	2.15–2.90	1.93	5.25	< 0.001
Medical students (*n* = 52)					
FTR	7.53	5.96–10.65	4.45	11.90	
SR	2.68	2.33–3.52	1.78	7.38	< 0.001
**FIRST DXA EXAM** (*n* = 48)					
Residents (*n* = 24)					
FTR	3.83	3.14–4.70	2.77	5.25	
SR	2.18	2.00–2.75	1.93	2.85	< 0.001
Medical students (*n* = 24)					
FTR	6.13	5.37–6.85	4.45	7.63	
SR	2.33	1.99–2.38	1.78	4.38	< 0.001
**FOLLOW-UP DXA EXAM** (*n* = 56)					
Residents (*n* = 28)					
FTR	6.07	5.20–6.70	4.47	7.45	
SR	2.85	2.58–3.14	2.17	3.14	< 0.001
Medical students (*n* = 28)					
FTR	9.93	8.55–11.27	5.88	11.90	< 0.001
SR	3.32	2.92–3.71	2.28	7.38	

Median reporting time, interquartile range (IQR), Minimum (Min.), Maximum (Max.), *P*-values for comparisons between free-text reports (FTRs) and structured reports (SRs) using Mann-Whitney U test

The difference in reporting time between SRs and FTRs was particularly pronounced for patients with more than one previous comparison. In these follow-up exams, residents had a 53.0% shorter reporting time for SRs than for FTRs (median for FTRs: 6.07 min; median for SRs: 2.85 min; *P* <  0.001) while the reduction for medical students amounted to 66.6% (median for FTRs: 9.93 min; median for SRs: 3.32 min; *P* <  0.001).

Comparison of reporting times of SRs created by residents vs. medical students showed no significant difference, either for all reports (*P* = 0.159) or for the two sub-groups (for single exams: *P* = 0.969; for follow-up exams: *P* = 0.060). Reporting times of FTRs created by residents vs. medical students were not compared since the residents used a free-speech dictation software while the medical students typed their reports.

### Report evaluation

Both FTRs and SRs were evaluated by referring physicians in terms of content, structure, comprehensibility and impact on clinical decision-making (see Table in Additional file 2):

### Content and appropriateness of report length

All eight pre-defined key features were included in the content of all FTRs and SRs. With respect to the report extent, all SRs were considered appropriate while only 79.2% of FTRs received the same rating. Among the remaining FTRs, 14.6% were rated as “too long” and 6.3% as “too short”. The percentage of FTRs found to have an appropriate extent was equal in both residents and medical students (79.2% in both). Yet, whereas all remaining FTRs by medical students were viewed as “too long” (20.9%), the ratings of the remaining FTRs by residents were mixed (“too short”: 12.5%, “too long”: 8.3%).

### Structure and comprehensibility

**Fig. 5:**
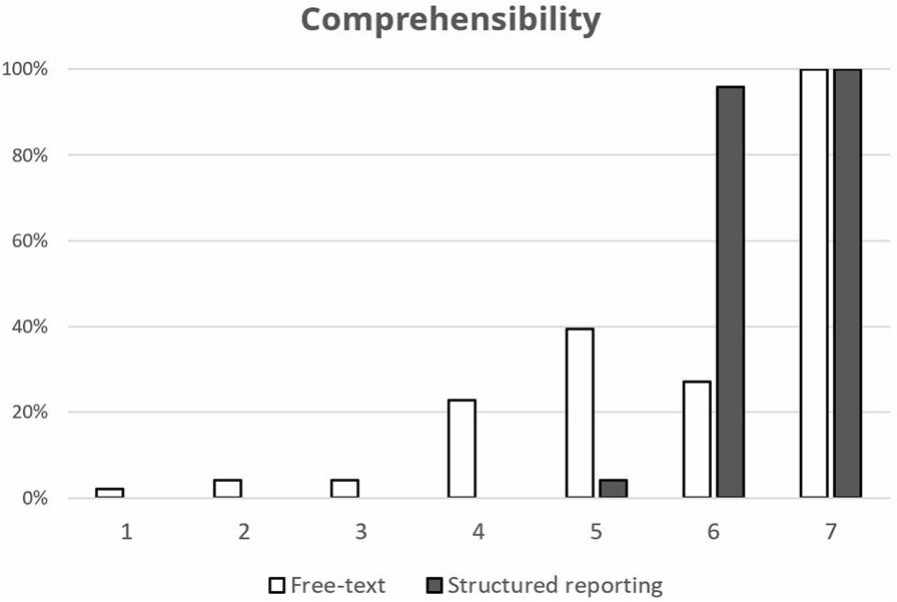
Comprehensibility: Ratings for comprehensibility were significantly higher for SR than for FTR

SRs were rated to be significantly superior to FTRs in the extraction of relevant information (*P* <  0.001). Also, the structure of SRs was found to be more helpful in terms of information gain than that of FTRs (*P* <  0.001), and ratings for comprehensibility were significantly higher for SRs (*P* <  0.001) (see Fig. 5).

### Clinical consequences

SRs were rated to answer the clinical question significantly better than FTRs (*P* <  0.01). SRs and FTRs showed no difference with respect to the contribution to the decision on further clinical management (*P* = 0.707). SRs received significantly higher ratings for the expression of a clear impression (*P* < 0.01).

### Overall quality ratings

**Fig. 6:**
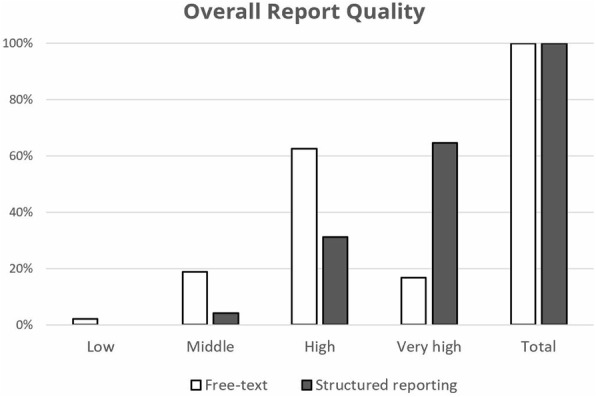
Overall report quality: SR received significantly higher ratings for overall report quality than FTR

Overall quality ratings were significantly higher for SRs vs. FTRs for both residents and medical students. In total, 64.6% of SRs received ratings of “very high quality” compared to only 16.7% of FTRs (difference: + 47.9%; *P* < 0.0001). Among the residents, there was an increase of “very high quality” ratings from 25% for FTRs to 58.3% for SRs (difference: + 33.3%; *P* = 0.0010). When comparing ratings by report type created by medical students, only 8.3% of FTRs were found to be of “very high quality” compared to 70.8% of SRs (difference: + 62.5%; *P* < 0.0001) (see Fig. 6).

### Preference of report type

One resident and both medical students stated structured reporting as their preferred reporting type for DXA examinations. Completeness, shorter reporting times and structure were viewed as the main strengths of SRs. Only the more experienced resident (40 months experience in radiology) preferred free-text reporting to structured reporting, explaining that he enjoyed free-speech dictation more than clicking and entering numbers, and that he did not like to use additional programs. Both evaluating clinicians preferred SRs to FTRs. The structure of SRs was perceived to reduce error rates and facilitate a quick extraction of relevant information.

## Discussion

### Template-based structured reports can significantly shorten reporting times and improve report quality

Our findings that SRs exhibit better completeness and result in higher satisfaction of referring physicians are consistent with and extend those from prior reports [6–13, 15, 16]. However, one common concern has been that structured reporting might be more time-consuming and complex than free-text reporting and thus impede productivity [18, 38].

Here we provide evidence that SRs may not only lead to better completeness and higher satisfaction of referring physicians but also save time, at least for highly standardized examination types such as DXA. Notably, the more experienced the subject was, the smaller the difference between SRs and FTRs became in terms of reporting time. This observation could be explained by the fact that the medical students typed their FTRs unlike the residents who used the free-speech dictation software. As typing takes more time than free-speech dictation our study might overestimate the difference between the reporting times for medical students and residents. Another explanation could be that residents were more efficient in generating FTRs as they had a more advanced level of experience.

One major advantage of structured reporting is that the user is less prone to make careless mistakes. In this study, for instance, the template not only indicated the reference ranges of the t-score in the decision tree (see Fig. 2), but also performed automatic calculations of the change of bone mineral density over time (in %).

Specific DXA report contents that may be missed or incorrectly reported in FTRs include the assessment of BMD changes across non-cross-calibrated machines, fracture risk and vertebral fracture assessment.

All in all, these findings have major medical and economic implications. In the light of the current demographic transition, the workload in radiology is rapidly increasing and structured reporting could make a relevant contribution to improving the efficiency of radiologic workflow. This is particularly true for DXA exams as the prevalence of osteoporosis is increasing in many countries [39–42]. More importantly, structured reporting of DXA exams can make a relevant contribution to improving patient outcome by providing the clinician with more comprehensible reports with higher quality.

### Structured reporting as an educational tool for residents and medical students

The template used in this study reveals that structured reporting can further be a powerful educational tool. Using info boxes within the user interface of the software, relevant background information and exemplary images and reports were displayed. In general, the info boxes might be particularly beneficial to illustrate anatomical images, classifications and up-to-date guidelines. This feature is highly useful for training inexperienced students and residents in a self-guided way. This theory is supported by our finding that the medical students preferred learning DXA reporting with a template and were able to generate very good SRs quickly. However, the medical students also reported that structured reporting might lead to a superficial evaluation due to just clicking through the template, which is a common concern about structured reporting [43]. In the present study, all four readers could easily utilize the software after a short initial training which indicates that only a minimum level of adaptation is required to switch from free-text reporting to structured reporting.

### Limitations

Despite the benefits of structured reporting over free-text reporting highlighted in this study, several limitations need to be acknowledged.

First, due to the retrospective nature of our study, our subjects created the reports in a study setting and not in actual clinical practice. Thus, findings will need to be validated using the template during routine clinical reporting.

Second, in clinical practice there is a broad spectrum of reports (FTR as well as SR) being created. For example, a survey of 265 radiologists in the United States found that only 51% used structured reporting for at least half of their reports [44]. Another survey in Italy found that 56% of radiologists never used structured reporting [17]. When it comes to bone density measurements, many centers are still creating FTRs while others have adopted templates like the ISCD’s which is essentially a form containing headings and sentences with blanks for the individual BMD, Z and T-Scores, among others [45]. Furthermore, fully structured online templates with more flexibility, like the one used in this study have been developed. These templates generate sentences automatically in standardized language depending on user entries. Other centers are even attempting to generate their DXA reports fully automatically, although they currently still require revision by radiologists [46]. Given these different approaches to structured reporting of bone density measurements with varying degrees of automation, it might also be beneficial to further evaluate and compare these different types of structured reporting.

Third, the number of subjects who created reports and their experience were limited. A prospective study including a more varied sample of reporting individuals is likely to provide further important insights into the potential of a widespread use of this template. One interesting hypothesis that could be tested is whether the time difference between FTRs and SRs decreases further with increasing experience, although our study indicates that the time required for SRs varies less with experience compared to FTRs.

Additionally, one may argue that the evaluation of report quality is rather subjective and is largely influenced by the evaluating clinician, potentially limiting the generalizability of our findings. Evaluations of the report quality by a larger, more diverse group of clinicians may be beneficial. However, due to the highly standardized nature of DXA exams, we believe that the quality ratings are likely to be consistent even among many referring physicians.

Finally, the observations of the present study cannot be generalized to other radiology examinations and their reports. Reporting times might be less likely to be improved by structured reporting in less standardized, highly variable exams, since a much more complex template structure would be required. But at the same time, reports of highly complex exams might especially benefit from the guidance of a template, since SRs were shown to exhibit higher completeness and allow better extraction of information [6–13]. Importantly, the extent to which structured reporting can improve reporting efficiency and quality strongly depends on the technical features of the utilized software. For instance, an automatic insertion of technical details into the radiology report was found to significantly improve report accuracy [47]. In a similar manner, features such as an automatic insertion of references to previous reports or an automatic identification of certain types of information could create added value, even for less standardized exam.

Further evaluation of different types of structured reporting templates in prospective (multicenter) studies with readers at various levels of experience and a larger number of evaluating physicians is likely to provide a broader impression.

## Conclusion

In highly standardized exams such as radiographic bone density measurements, template-based structured reporting might lead to shorter reporting times. At the same time, structured reporting may improve report quality and serve as an effective educational tool for medical students and radiology residents during their training.

## Supplementary information

**Additional file 1.** Reporting times for DXA exams.

**Additional file 2.** Report evaluation by referring clinicians.

**Additional file 3.** Reader’s survey.

## Data Availability

The datasets used and/or analysed during the current study are available from the corresponding author on reasonable request.

## Abbreviations

DXA: Dual X-ray absorptiometry
FTR: Free-text report
SR: Structured report
ISCD: The International Society for Clinical Densitometry
